# The short- and long-term outcome after the surgical management of common bile duct stones in a tertiary referral hospital

**DOI:** 10.1007/s00423-023-03011-2

**Published:** 2023-07-29

**Authors:** Gabriel F. Hess, Philipp Sedlaczek, Jasmin Zeindler, Simone Muenst, Andreas M. Schmitt, Silvio Däster, Martin Bolli, Otto Kollmar, Savas D. Soysal

**Affiliations:** 1 Clarunis, University Centre for Gastrointestinal and Liver Diseases, 4002 Basel, Switzerland; 2 Faculty of Medicine, University of Basel, Klingelbergstrasse 61, 4056 Basel, Switzerland; 3 Institute of Medical Genetics and Pathology University Hospital Basel, Schönbeinstrasse 40, 4056 Basel, Switzerland; 4 Department of Internal Medicine, Medical Oncology, University Hospital Basel, Petersgraben 4, 4051 Basel, Switzerland

**Keywords:** Common bile duct stones, Open choledochotomy, Hepaticojejunostomy, Recurrent common bile duct stones, Acute cholangitis

## Abstract

**Background:**

The removal of common bile duct stones by endoscopic retrograde cholangiopancreatography (ERCP) shows excellent results with low complication rates and is therefore considered a gold standard. However, in case of stones non-removable by ERCP, surgical extraction is needed. The surgical approach is still controversial and clinical guidelines are missing. This study aims to analyze the outcomes of patients treated with choledochotomy or hepaticojejunostomy for common bile duct stones.

**Methods:**

All patients who underwent choledochotomy or hepaticojejunostomy for common bile duct stones at a tertiary referral hospital over 11 years were included. The analyzed data contains basic demographics, diagnostics, surgical parameters, length of hospitalization, and morbidity and mortality.

**Results:**

Over the study period, 4375 patients underwent cholecystectomy, and 655 received an ERCP with stone extraction, with 48 of these patients receiving subsequent surgical treatment. ERCP was attempted in 23/30 (77%) of the choledochotomy patients pre/intraoperatively and 11/18 (56%) in hepaticojejunostomy patients. The 30-day major complication rate (Clavien-Dindo > II) was 1/30 (3%) in the choledochotomy group and 2/18 (11%) in the hepaticojejunostomy group. Complications after 30 days occurred in 3/30 (10%) patients and 2/18 (11%), respectively, and no mortality occurred.

**Conclusion:**

ERCP should still be considered the gold standard, although due to low short- and long-term morbidity rates, choledochotomy and hepaticojejunostomy represent effective surgical solutions for common bile duct stones.

**Supplementary Information:**

The online version contains supplementary material available at 10.1007/s00423-023-03011-2.

## Introduction

Common bile duct stones (CBDS) are present in about 10–20% of patients with symptomatic gallstones [1–4]. Occlusion of the common bile duct (CBD) can lead to pathologic liver function tests with a cholestatic picture and complications such jaundice, cholangitis, liver abscesses, or pancreatitis [5, 6]. Recurrence of CBDS is the most common long-term complication after gallstone surgery, and patients usually present with acute cholangitis [7, 8].

Since the National Institutes of Health Consensus Conference in 1993, laparoscopic cholecystectomy is the current gold standard for gallstone disease [9]. In obstructive cholelithiasis, ERCP with endoscopic sphincterotomy as well as laparoscopic surgery has replaced open cholecystectomy (CCY) with choledochotomy or sphincterotomy in combination with bile duct clearance [10, 11]. In modern days with significant advances of the endoscopic standard techniques, CBDS clearance rates above 80% should be expected. In combination with further advanced techniques such as mechanical lithotripsy, success rates increased to more than 90%. In the remaining cases, additional specific endoscopic procedures (i.e., electrohydraulic, laser, or extracorporeal shockwave lithotripsy) or surgery is needed [12].

Apart from the gold standard of ERCP and laparoscopy [13, 14], several studies describe open cholecystectomy with bile duct exploration as a safe and feasible alternative in challenging cases [15–19]. Interestingly, some studies specify open choledochotomy as superior in clearing CBDS without a negative impact on morbidity or mortality [20–22].

Furthermore, Roux-en-Y hepaticojejunostomy (HJS) is a surgical modality for the treatment of recurrent CBDS after unsuccessful interventional therapy to allow bile drainage [23, 24].

Clinical guideline recommendations for the surgical management of recurrent CBDS are missing [24]. Therefore, the aim of this study was to describe the short- and long-term outcomes as well as the recurrence of CBDS after surgical management with choledochotomy and HJS.

## Material and methods

In this retrospective single-center analysis, we included all patients who underwent choledochotomy or HJS for CBDS between 2009 and 2020 at St. Clara Hospital in Basel, Switzerland. Additionally, total numbers of CCY and acute cholecystitis cases were documented. Patient records of hospitalization for choledochotomy/HJS and all relevant clinical follow-up information concerning recurrence, surgery, or death were compiled. The data included demographics such as age, gender, previous abdominal surgeries, details about the choledochotomy and HJS procedure, complications, duration of total hospital stay, revisional surgery and/or reintervention rate, morbidity, and mortality [25, 26]. Complications were classified according to the Clavien-Dindo (CD) classification and divided into short- and long-term complications [27–29]. In this study, we defined short-term complications within a period of 30 days postoperative and long-term complications after 30 days. Follow-up data was collected through patient’ readmission at St. Clara Hospital Basel or University Hospital Basel in any subspecialty unit. Furthermore, preoperative ERCP were analyzed. The total amount of CCY and ERCP over the same period served as a reference value. Reporting followed the Strengthening the Reporting of Observational Studies in Epidemiology (STROBE) reporting guideline where appropriate [30].

Data were extracted retrospectively in dedicated electronic databases (CGM PHOENIX) by trained contributors with regular auditing and guidance by the principal investigator from a prospective consecutive institutional database (Microsoft Office created ACCESS-database, version 14.0.7015. 1000, Office 2013; Microsoft Corporation, Redmond, Washington, USA).

We used descriptive statistics to summarize patient, treatment, and disease characteristics. Baseline differences between the two groups were calculated using the *χ*^2^ test, the *t*-test, or the Welch test as appropriate. Data are shown as mean ± SD. Ethical approval was granted by the ethics commission of North West Switzerland with the registration number *EKNZ 2020–00076*.

## Results

In the reported time period of 11 years, 4375 patients underwent CCY, and 1801 patients received ERCP, of which 655 had a stone extraction. 48 patients underwent surgery for open bile duct exploration because of CBDS, resulting in either choledochotomy (30; 63%) or hepaticojejunostomy (18; 37%). Further information about the patient characteristics at the time of the first presentation can be found in Table 1.

**Table 1:** Baseline characteristics

	All patients	Choledochotomy	HJS	*p* value
	*N* = 48	*N* = 30	*N* = 18	
Age, years—mean (SD)	71 (14)	75 (10)	66 (17)	0.03
Sex, female—*n* (%)	23 (48)	16 (53)	7 (39)	0.50
Length admission, days—mean (SD)	20 (7)	20 (4)	21 (10)	0.66
Apache II score—mean (SD)	8 (2)	8 (2)	7 (3)	0.20
Recurrent cholelithiasis—*n* (%)	23 (48)	11 (37)	12 (67)	0.09
ERCP—*n* (%)	35 (73)	23 (77)	12 (67)	0.68
Before surgery	32 (67)	21 (70)	11 (61)	–
During surgery	1 (2)	0 (0)	1 (6)	–
Complications	1 (2)	1 (3)	0 (0)	–
Previous abdominal surgery—*n* (%)	35 (73)	20 (67)	15 (83)	0.36
Comorbidities—*n* (%)				
Significant comorbidities	45 (94)	28 (93)	17 (94)	1.00
None	3 (6)	2 (7)	1 (6)	1.00

*SD* standard deviation, *CI* confidence interval Apache II Score [31]

Surgery time was significantly longer in the HJS group with a mean time of 291 ± 72 min (95% CI: 219–363) compared to 174 ± 25 min (95% CI: 149–199) in the choledochotomy group (*p* value < 0.01). In the HJS group, the gallbladder was removed prior to bile duct resection in 13/18 cases (72%) whereas in the choledochotomy group this was the case in 8/30 patients (27%) (*p* value = 0.01).

Within 30 days after surgery, 16/48 patients (33%) needed treatment due to complications. Grade I and II complications occurred in 13/48 (25%) patients, grade IIIa and IIIb in three patients (6%), and one patient (2%) suffered a grade IVa complication and had to be transferred to intensive care unit. One patient needed postoperative abscess drainage, in one patient the HJS had to be refashioned after 10 days, and one patient developed postoperative heart failure. There was no reported mortality within the first 30 postoperative days.

5/48 patients (10%) were readmitted > 30 days after surgery, three (10%) in the choledochotomy group and two (11%) in the HJS group. All three complications in the choledochotomy group were due to recurrent cholelithiasis and could be treated with ERCP. In the HJS group, one patient (5.6%) presented to our department again after 295 days due to recurrent choledocholithiasis and was treated by drainage and removal of the bile duct stone through balloon extraction. In one patient, recurrent cholangitis occurred after more than two years and was treated conservatively with antibiotics. Interestingly, one patient initially presented with an existing HJS which had been done 23 years earlier in a different hospital. A stricture could be identified radiologically, and new HJS with further bile duct resection was done (Tables 2 and 3).

**Table 2:** Surgical information

	All patients	Choledochotomy	HJS	*p* value
	*N* = 48	*N* = 30	*N* = 18	
Surgery time, minutes—mean (SD)	212 (86)	174 (51)	291 (94)	< 0.01
Cholecystectomy—*n* (%)				
Before surgery	21 (44)	8 (27)	13 (72)	0.01
During surgery	27 (56)	22 (73)	5 (28)	0.01
Bile duct revision prior to HJS—*n* (%)				
During CCY	4 (8)	–	4 (22)	–
Laparoscopic try—*n* (%)	5 (10)	3 (10)	2 (11)	1.00
Stone diameter—mean (SD)	1.4 (0.8)	1.5 (0.8)	1.3 (0.8)	0.78
Successful clearance—*n* (%)	48 (100)	30 (100)	18 (100)	–
Decision for HJS—(%)				
Primary	5 (10)	–	5 (28)	–
Post-CCY—complication	4 (8)	–	4 (22)	–
Post-CCY—recurrent cholangitis	9 (19)	–	9 (50)	–
Emergency setting—*n* (%)	9 (19)	5 (17)	4 (22)	0.92

*HJS* hepaticojejunostomy, *CCY* cholecystectomy

**Table 3:** Complications according to Clavien/Dindo (CD). Minor complications (I–II), major complications (> III)

	All patients	Choledochotomy	HJS	*p* value
	*N* = 48	*N* = 30	*N* = 18	
CD within the first 30 days	16 (33)	8 (27)	8 (44)	0.343
I	5 (10)	2 (7)	3 (17)	
II	7 (15)	5 (17)	2 (11)	
IIIa	2 (4)	1 (3)	1 (6)	
IIIb	1 (2)	0 (0)	1 (6)	
IV	1 (2)	0 (0)	1 (6)	
CD after 30 days or later	5 (10)	3 (10)	2 (11)	1.00
ERCP post-OP	4 (8)	4 (13)	0 (0)	0.28
Stone extraction < 30 days	1 (2)	1 (3)	0 (0)	1.00
Stone extraction > 30 days	3 (6)	3 (10)	0 (0)	0.44
Recurrent CBDS	4 (8)	3 (10)	1 (6)	1.00

*CD* Clavien/Dindo

## Discussion

Minimally invasive methods such as ERCP have been well established as the gold standard for the treatment of CBDS. However, even with an increase of reliability over the past decades, some CBDS cannot be successfully treated with ERCP. This study demonstrates that choledochotomy and HJS are both well-established methods for the management of CBDS. Even though choledochotomy and HJS are usually used in highly complicated cases of CBDS, complication rates are relatively low and represent a valid alternative to laparoscopic bile duct surgery. Based on our findings, shorter operation times, lower short-term complication rates, a sufficient long-term outcome considering CBDS recurrence, and additional surgical options seem to be the main advantages of choledochotomy over the establishment of a hepaticojejunostomy.

The study shows that open surgery such as choledochotomy for complex bile duct stones is a rare procedure. This is in line with the guidelines of using ERCP as the gold standard and shows that surgery was only suggested if previous interventions failed or were not possible [13, 14, 32].

Taking into account our results, open surgery can be considered a safe and feasible procedure in the treatment of CBDS, even in a hospital without a specialized hepatobiliary surgeon. It has to be noted that training can be challenging, since with the advances in ERCP procedures case numbers have become rare. Consequently, the method of choice should be based on the preference and experience of the operating surgeon.

Comparing choledochotomy with HJS shows that complications within 30 days after surgery are more frequent in the HJS group. Long-term complications after more than 30 days postsurgery as well as recurrence rates of CBDS were similar throughout both study groups. In line with these results, the current literature describes complication rates between 3 and 41% for open choledochotomy, with a trend toward lower rates after primary closure compared to T-tube-insertion [33–38]. When consulting literature concerning the overall short-term complication rates for HJS, numbers between 9 and 29% are found. Mortality is described in up to 3% [24, 39]. To our knowledge, this study seems to be the first study in which the short- as well as the long-term complications of HJS compared to open choledochotomy specifically for CBDS are systematically investigated.

Regardless of the excellent stone clearance rates with modern endoscopy, several authors report a recurrence rate between 3 and 15% of CBDS after an initially successful endoscopic clearance [24, 40–42]. Xia et al. observed that the main reason for CBDS recurrences seems to be related to structural and functional abnormalities or damages of the duodenal papilla (sphincter Oddi). This can be caused by interventional incision of the duodenal papilla as it is performed in a sphincterotomy during ERCP [24]. Furthermore, repeated ERCP procedures have been shown to increase the complication rate during the intervention [39].

In their study, Abdelmajid et al. have defined main indicators for biliary enteric bypass: age > 65 years (90% of the cases), multiple stones (55%), and unremovable stones (16%) [23]. In our study population, main reasons for establishing a HJS were recurrent symptoms of obstructive bile duct disease, such as CBDS, choledochal stenosis/sclerosis, and complications occurring during treatment. Interestingly, based on the presented data and the literature, HJS seems to be a widely adoptable method for terminal bile duct disease or challenging anatomic sites even though it is a more invasive procedure [23, 43, 44]. Ultimately, it has to be noted that even though there are some indicators pointing toward the establishment of an HJS rather than to perform a choledochotomy, the final decision should be the surgeon’s preference.

HJS was technically successful in all of the cases in our cohort. Despite its technical success, it should be noted that open biliodigestive anastomoses inhibit any further efforts for minimal invasive treatment of CBDS and are therefore considered as a last option for CBDS, pre- or post-CCY [6]. Additionally, since the resection line of the bile duct should be as high as possible to decrease the risk for bile duct stricture, there are very limited options in case the HJS fails [45].

According to the literature, strictures are the most frequent long-term complications of HJS, resulting in bile duct obstruction potentially causing recurrent cholangitis [45–51]. Commonly, stricture rates range between 5 to 14%, are usually diagnosed around 17 to 18 months after surgery, and are mostly caused by scar-tissue contraction [49, 50]. Consequently, the higher the biliodigestive anastomosis is placed, the lower the possibility of stricture formation [45, 52, 53].

This study has several limitations. First of all, this is a retrospective study and data was not documented for research purpose, so potential confounders might be missing. Secondly, this is a single-center study; therefore, the outcome might be biased on the surgical team’s skill and experience of the procedure. Additionally, there are only few patients with choledochotomy or HJS for CBDS, and statistics are only descriptive. Lastly, the loss to follow-up was quite high, possibly due to the tertiary hospital with an outsourced aftercare. Fixed follow-up appointments or reports could have been helpful. Further research with a higher number of patients is needed to fully investigate the best use of either choledochotomy or HJS and their outcomes.

## Conclusion

This study has demonstrated that when ERCP does not show sufficient results for the management of CBDS, choledochotomy and HJS can both be considered a safe and effective procedure with low morbidity and mortality. With shorter operation times, lower short-term complication rates, a sufficient long-term outcome considering the CBDS recurrence, and further surgical options, choledochotomy seems to be the method of choice for choledocholithiasis. However, HJS plays a role in cases with complications after CCY or ERCP and can be discussed as a method of first choice for recurrent bile stones due to its possible positive effect on the formation of recurrent CBDS.

### Supplementary Information

Below is the link to the electronic supplementary material.

Supplementary file1 (DOCX 537 KB)

## Data Availability

The data that support the findings of this study are available on request from the corresponding author, [SDS]. The data are not publicly available due to [ethical restrictions].

## Abbreviations

APACHE: Acute physiology and chronic health evaluation
CBDS: Common bile duct stones
CCI: Charlson Comorbidity Index
CD: Clavien-Dindo
CCY: Cholecystectomy
CI: Confidence interval
ERCP: Endoscopic retrograde cholangiopancreatography
HJS: Hepaticojejunostomy
SD: Standard deviation
