# Supplementary material for: The short- and long-term outcome after the surgical management of common bile duct stones in a tertiary referral hospital
Source: Langenbecks Arch Surg. 2023 Jul 29;408(1):288. doi: 10.1007/s00423-023-03011-2 (PMC10386922; doi:10.1007/s00423-023-03011-2)
Supplement: Supplementary file 1 — Supplementary file1 (DOCX 537 KB) [file 423_2023_3011_MOESM1_ESM.docx]

**Surgical procedure**

Hereafter recommend details about the procedure. Firstly, the incision for the choledochotomy should be made perpendicularly to the ductus hepatocholedochus (DHC). Although, in the surgical reports of this study, the type of the choledochotomy incision (perpendicular or longitudinal) was not reported. Secondly, the incision should be made in the middle third of the CBD. Thirdly, intraoperative imaging, such as cholangiography or cholangioscopy, should be performed. Fourthly, the T-tube should be placed through a separate and smaller incision more proximal to the liver to enable optimal healing and to enable an easy possibility for follow-up cholangiography. Finally, an Easy-flow drainage should be placed behind the primary incision to indicate relevant biliary leakage after T-tube removal. The latter should take place after seven days.

Figure 1: Surgical Procedure of an open choledochotomy

| 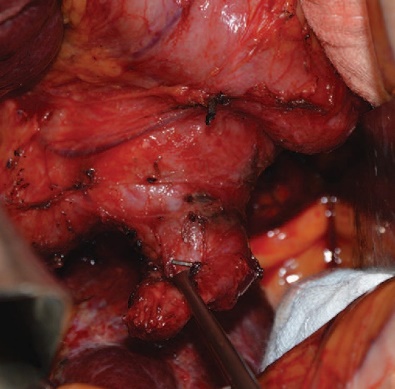A: Cystic stump grasped by a clamp | 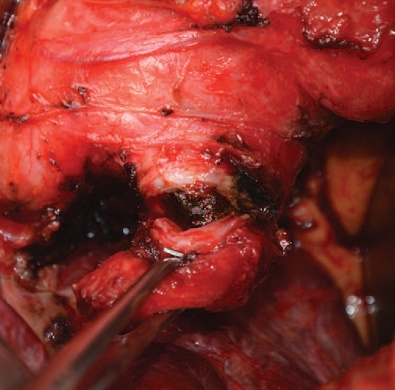B: Perpendicular choledochal incision | 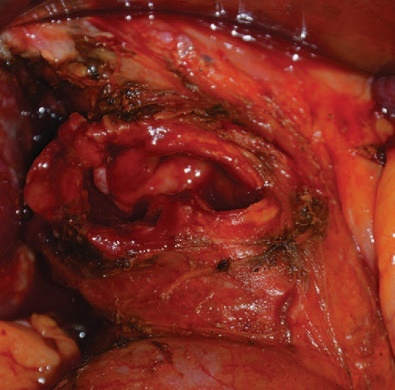C: Lumen after stone removal |
| --- | --- | --- |
| D: Preparation of the monofilament non-absorbable sutures 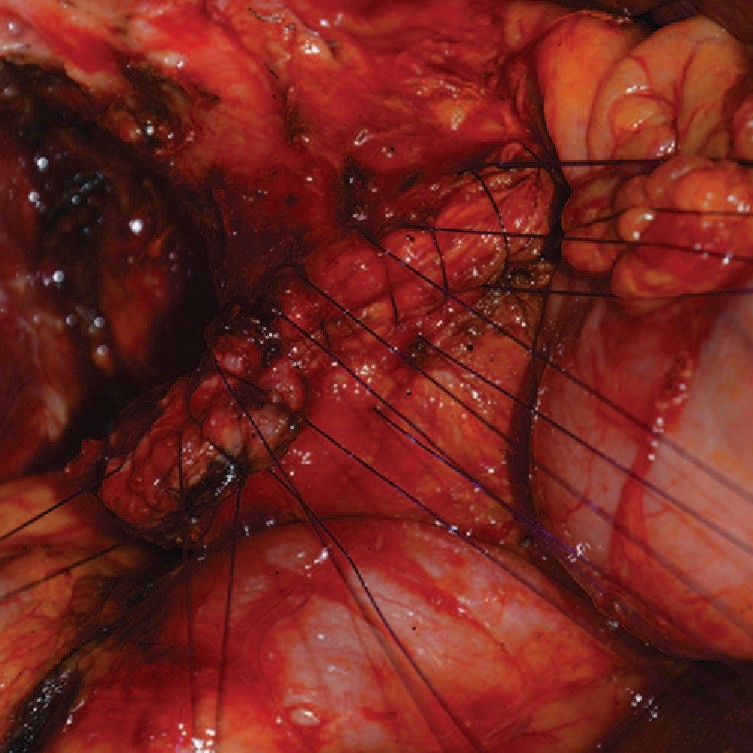 | E: Knotted sutures 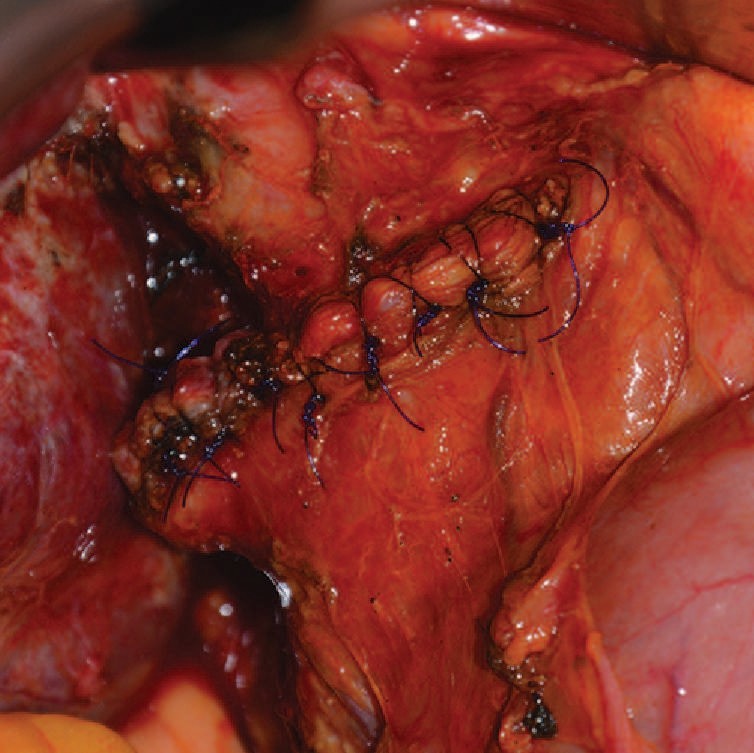 |  |
| 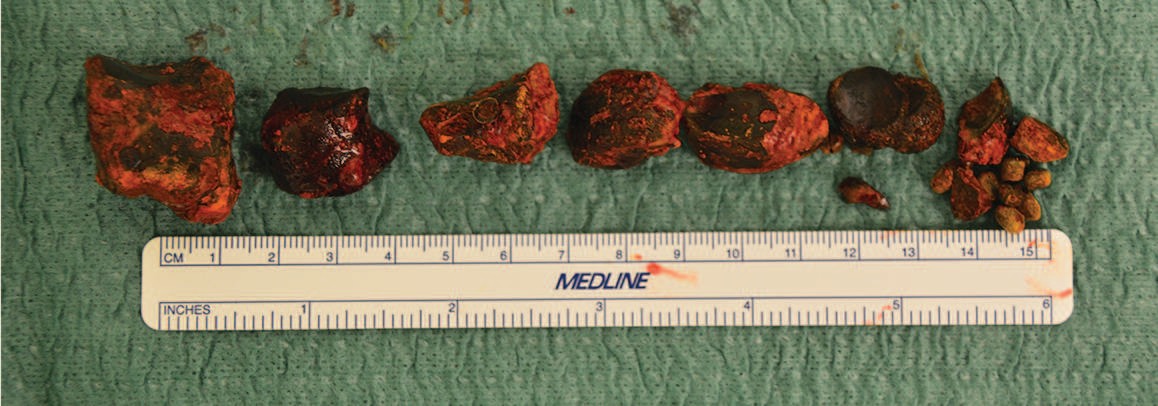 | | |
| F: extracted cholelithiasis |  |  |
